# Supplementary material for: Potential Clinical Impact of LAFOV PET/CT: A Systematic Evaluation of Image Quality and Lesion Detection
Source: Diagnostics (Basel). 2023 Oct 24;13(21):3295. doi: 10.3390/diagnostics13213295 (PMC10650426; doi:10.3390/diagnostics13213295)
Supplement: Supplementary file 1 [file diagnostics-13-03295-s001.zip › Supplementary materials.pdf]

Supplementary materials

Table S1: Referral texts

List of provided referral texts for the patients otherwise blinded during lesion detection

1; T.C. Cervical cancer	35; P.D. Ovarian cancer	69; R. Hodgkins lymphoma
2; T.C. Cervical cancer	36; P.D. Ovarian cancer	70; R. Hodgkins lymphoma
3; T.C. Cervical cancer	37; P.D. Ovarian cancer	71; R. Hodgkins lymphoma
4; T.C. Cervical cancer	38; P.D. Ovarian cancer	72; R. Lymphoma
5; T.C. Cervical cancer	39; P.D. Ovarian cancer	73; R. Lymphoma
6; T.C. Esophageal cancer	40; P.D. Lung cancer	74; R. Lymphoma
7; T.C. Hepatic cancer	41; P.D. Vulvar cancer	75; R. Lymphoma
8; T.C. Disseminated breast cancer	42; P.D. Lymphoma	76; R. Melanoma
9; T.C. Pancreatic cancer	43; P.D. Lymphoma	77; R. Melanoma
10; T.C. Lung cancer (mesothelioma)	44; P.D. Sacral tumor	78; R. Melanoma
11; T.C. Bladder cancer	45; P.D. Occult primary tumor	79; R. Melanoma
12; T.C. Vulvar cancer	46; P.D. Ovarian cancer	80; R. Peritoneal mesothelioma
13; T.C. Vulvar cancer	47; P.D. Vulvar cancer	81; R. Myeloma
14; T.C. Hepatocellular carcinoma	48; P.D. Occult primary tumor	82; R. Lung cancer (NSCLC)
15; T.C. Melanoma	49; P.D. Melanoma	83; R. Lymphoma (PMBCL)
16; T.C. Melanoma	50; R. Cervical cancer	84; S. Cardiac cancer
17; T.C. Lung cancer (NSCLC)	51; R. Cervical cancer	85; S. Cervical cancer
18; T.C. Lung cancer (NSCLC)	52; R. Cervical cancer	86; S. Cervical cancer
19; T.C. Lung cancer (mesothelioma)	53; R. Cervical cancer	87; S. Cervical cancer
20; P.D. Breast cancer	54; R. Cervical cancer	88; S. Cervical cancer
21; P.D. Ovarian cancer	55; R. Colon cancer / Breast cancer	89; S. Cervical cancer
22; P.D. Cervical cancer	56; R. Hepatic cancer	90; S. Uterine cancer
23; P.D. Uterine cancer	57; R. Breast cancer	91; S. Breast cancer
24; P.D. Uterine cancer	58; R. Breast cancer	92; S. Breast cancer
25; P.D. Uterine cancer	59; R. Breast cancer	93; S. Breast cancer
26; P.D. Uterine cancer	60; R. Breast cancer	94; S. Breast cancer
27; P.D. Breast cancer	61; R. Ovarian cancer	95; S. Breast cancer
28; P.D. Breast cancer	62; R. Ovarian cancer	96; S. Breast cancer
29; P.D. Breast cancer	63; R. Ovarian cancer	97; S. Breast cancer
30; P.D. Esophageal cancer	64; R. Pancreatic cancer	98; S. Penile cancer
31; P.D. Occult primary tumor	65; R. Urinary cancer	99; S. Fallopian tube cancer
32; P.D. Ovarian cancer	66; R. Vaginal cancer	100; S. Vulvar cancer
33; P.D. Ovarian cancer	67; R. Vulvar cancer	
34; P.D. Ovarian cancer	68; R. Chronic lymphocytic leukemia	

T.C: Treatment control, P.D: Primary diagnostics, R: Rrecurrence, S:Staging

Table S2: Pre-defined lists of organ sites, lymph nodes and anatomical regions

**Organ**
1. Brain
2. Oro/naso/hypopharynx/larynx
3. Thyroid
4. Lung, right
5. Lung, left
6. Pleura, right
7. Pleura, left
8. Breast, right
9. Breast, left
10. Oesophagus
11. Liver
12. Pancreas
13. Adrenal, right
14. Adrenal, left
15. Kidney, right
16. Kidney, left
17. Spleen
18. Stomach
19. Bowel
20. Peritoneal
21. Ovary, right
22. Ovary, left
23. Uterus
24. Cervix
25. Vagina/vulva
26. Prostate
27. Penis/scrotum
28. Bones
29. Cutis
30. Other
**Lymphnodes**
31. Head/neck, right
32. Head/neck, left
33. Axilla/subpect, right
34. Axilla/subpect, left
35. Mediastinum/hilar/retrocrural
36. Int mam, precardiac
37. Retroperitoneal
38. Abdominal
39. Iliac, right
40. Iliac, left
41. Groin, right
42. Groin, left
43. Lnn, other

**Ear Nose Throat region**
Organ
2. Oro/naso/hypopharynx/larynx
3. Thyroid
Lymph nodes
31. Head/neck, right
32. Head/neck, left
**Thoracic region**
Organ
4. Lung, right
5. Lung, left
6. Pleura, right
7. Pleura, left
10. Esophagus
Lymph nodes
35. Mediastinum/hilar/retrocrural
**Abdominal**
Organ
11. Liver
12. Pancreas
13. Adrenal, right
14. Adrenal, left
15. Kidney, right
16. Kidney, left
17. Spleen
18. Stomach
19. Bowel
20. Peritoneal
Lymph nodes
37. Retroperitoneal
38. Abdominal
**Pelvic region**
Organ
21. Ovary, right
22. Ovary, left
23. Uterus
24. Cervix
25. Vagina/vulva
26. Prostate
27. Penis/scrotum
Lymph nodes
39. Iliac, right
40. Iliac, left

Figure S1: Total image findings

Patient	30 sek				90 sek				180 sek				300 sek				600 sek			
Organ	Count (0-5	Benign* (	Equivocal	Malignan	Count (0-5	Benign* (	Equivocal	Malignan	Count (0-5	Benign* (	Equivocal	Malignan	Count (0-5	Benign* (	Equivocal	Malignan	Count (0-5	Benign* (	Equivocal	Malignan
Brain	1	1	0	0	1	1	0	0	1	1	0	0	1	1	0	0	1	1	0	0
Oro/naso/hypopharynx/larynx	17	15	0	2	23	20	1	2	24	21	1	2	23	20	1	2	23	20	1	2
Thyroid	15	6	8	1	16	8	7	1	17	10	6	1	17	10	6	1	17	10	6	1
Lung, right	23	12	3	8	25	13	3	9	25	14	1	10	23	13	1	9	23	12	2	9
Lung, left	16	6	4	6	18	9	3	6	18	9	3	6	18	9	3	6	18	9	3	6
Pleura, right	13	1	5	7	15	2	6	7	15	2	6	7	15	2	6	7	15	2	6	7
Pleura, left	9	1	0	8	9	1	1	7	9	1	1	7	9	1	1	7	9	1	1	7
Breast, right	17	1	3	13	16	1	1	14	16	1	1	14	17	1	2	14	18	1	3	14
Breast, left	15	1	4	10	17	1	6	10	17	1	6	10	17	3	4	10	17	3	4	10
Oesophagus	8	3	4	1	9	6	2	1	10	7	2	1	10	7	2	1	10	7	2	1
Liver	18	1	5	12	18	0	4	14	17	1	0	16	19	1	2	16	21	2	3	16
Pancreas	2	0	0	2	2	0	0	2	2	0	0	2	2	0	0	2	2	0	0	2
Adrenal, right	2	1	0	1	2	1	0	1	2	1	0	1	2	1	0	1	2	1	0	1
Adrenal, left	2	2	0	0	3	3	0	0	4	4	0	0	2	2	0	0	2	2	0	0
Kidney, right	0	0	0	0	0	0	0	0	0	0	0	0	0	0	0	0	0	0	0	0
Kidney, left	0	0	0	0	0	0	0	0	0	0	0	0	0	0	0	0	0	0	0	0
Spleen	2	0	0	2	2	0	0	2	6	0	0	6	6	0	0	6	6	0	0	6
Stomach	3	1	1	1	6	3	2	1	6	3	2	1	6	4	1	1	6	4	1	1
Bowel	40	23	12	5	45	33	7	5	45	33	7	5	47	35	7	5	47	35	7	5
Perotoneal	26	1	2	23	26	1	2	23	29	3	1	25	28	4	0	24	29	4	0	25
Ovary, right	11	6	3	2	11	7	2	2	11	7	2	2	11	8	1	2	11	8	1	2
Ovary, left	11	8	3	0	11	9	2	0	11	9	2	0	11	9	2	0	11	9	2	0
Uterus	10	3	1	6	9	2	1	6	10	3	1	6	10	3	1	6	10	3	1	6
Cervix	10	2	1	7	12	3	2	7	12	3	2	7	12	3	2	7	11	2	2	7
Vagina/vulva	8	4	2	2	9	4	3	2	9	5	1	3	9	5	1	3	8	4	1	3
Prostate	3	1	2	0	3	1	1	1	5	2	2	1	5	3	1	1	5	3	1	1
Penis/scrotum	2	2	0	0	2	2	0	0	4	4	0	0	5	5	0	0	5	5	0	0
Bones	52	21	6	25	60	32	2	26	61	33	2	26	63	37	2	24	63	37	2	24
Cutis	22	16	1	5	23	16	2	5	24	17	2	5	24	18	1	5	23	17	1	5
Other	94	78	5	11	102	85	4	13	103	85	1	17	104	85	2	17	103	85	2	16
Total, organs	452	217	75	160	495	264	64	167	513	280	52	181	516	290	49	177	516	287	52	177
Lymphnodes	Count (0-5	Benign* (	Equivocal	Malignan	Count (0-5	Benign* (	Equivocal	Malignan	Count (0-5	Benign* (	Equivocal	Malignan	Count (0-5	Benign* (	Equivocal	Malignan	Count (0-5	Benign* (	Equivocal	Malignan
Head/neck, right	34	24	0	10	35	25	0	10	42	31	1	10	48	37	1	10	49	39	0	10
Head/neck, left	55	33	6	16	57	32	5	20	68	44	4	20	72	48	4	20	73	52	1	20
Axilla/subpect, right	59	26	12	21	71	35	10	26	80	43	8	29	90	53	7	30	91	54	7	30
Axilla/subpect, left	76	36	14	26	89	60	2	27	97	68	2	27	102	74	1	27	107	80	0	27
Mediastinum/hilar/retrocrural	42	4	8	30	51	16	2	33	56	21	1	34	59	23	2	34	62	26	1	35
Int mam, precardiac	13	0	1	12	18	0	2	16	20	1	0	19	21	1	1	19	21	1	0	20
Retroperitoneal	40	3	7	30	42	4	7	31	43	4	7	32	43	4	7	32	43	3	7	33
Abdominal	17	1	0	16	17	1	0	16	20	1	1	18	22	4	0	18	19	1	0	18
Iliac, right	29	0	5	24	32	5	2	25	35	7	2	26	37	9	2	26	37	10	2	25
Iliac, left	28	1	3	24	30	3	2	25	32	5	1	26	34	8	0	26	36	7	4	25
Groin, right	24	6	4	14	26	9	3	14	35	20	1	14	47	32	1	14	47	32	1	14
Groin, left	21	7	3	11	26	11	4	11	35	20	4	11	46	32	3	11	45	31	3	11
Lnn, other	22	2	2	18	23	2	2	19	23	2	2	19	23	2	2	19	25	2	2	21
Total, nodes	460	143	65	252	517	203	41	273	586	267	34	285	644	327	31	286	655	338	28	289
